# Effect of Brick Aggregate Content on Performance of Recycled Construction-Solid-Waste Aggregate

**DOI:** 10.3390/ma17112616

**Published:** 2024-05-29

**Authors:** Xuan Zhu, Le Ding, Yuexing Wu, Xinzhong Wang, Xianliang Tan

**Affiliations:** 1 School of Civil Engineering, Hunan City University, Yiyang 413000, China; dingle@hncu.edu.cn (L.D.); wuyuexing@hncu.edu.cn (Y.W.); wxz811@hncu.edu.cn (X.W.); tanxianliang@hncu.edu.cn (X.T.); 2 National Engineering Research Center for Highway Maintenance Technology, Changsha University of Science & Technology, Changsha 410114, China

**Keywords:** construction solid waste, recycled aggregate, brick slag content, subgrade

## Abstract

In road engineering, road construction requires a large amount of natural aggregate; its substitution with recycled construction-solid-waste aggregate not only saves resources but also reduces the burden on the environment. The main components of construction solid waste are concrete blocks and brick slag; the breakability of the latter can affect the performance of mixed recycled aggregate, which hinders the use of construction solid waste in road engineering applications. To analyze the applicability of recycled construction-solid-waste aggregate containing brick slag aggregate in the subgrade layer, the effect of brick aggregate content on the CBR (California bearing ratio) and crushing value of mixed recycled aggregates was evaluated based on laboratory tests, and the field compaction quality of the recycled aggregates was analyzed. The results show that the 9.5–19 mm mixed recycled aggregate samples were crushed to a higher degree during the compaction process. A brick aggregate content less than 40% had little effect on the performance of mixed recycled construction-solid-waste aggregate. It is recommended to use a 22 t road roller for five passes (two weak vibrations + two strong vibrations + one weak vibration) at a speed of 3 km/h in the main compaction stage of the subgrade filling.

## 1. Introduction

### 1.1. Research Background

With the development of urbanization, the production of construction solid waste has increased rapidly, which has put enormous pressure on the environment [1]. The traditional method for construction-solid-waste treatment is landfill, which not only occupies a large amount of land but is also harmful to the environment and human health [2,3]. In contrast, the utilization of renewable resources is an important process in clean production, and the utilization of construction waste could achieve sustainable development and environmental protection in the construction industry [4,5].

Construction solid waste is difficult to use directly due to its large volume. Therefore, in order to improve its utilization efficiency, it is generally crushed and recycled into aggregate [6,7]. In structural engineering, there are strict requirements for mechanical performance; as the strength of subgrade materials is relatively low [8,9,10], the application of recycled construction-solid-waste aggregates for subgrade filling is one of the ways to efficiently utilize waste-based resources [11].

Recycled construction-solid-waste aggregate mainly includes recycled concrete aggregate and brick slag aggregate [12]. There are bottlenecks in the development of technology for the separation of the two; therefore, the recycling and utilization of brick–concrete mixed aggregates are particularly important [13,14]. The hardness of recycled brick aggregate from broken brick slag is low, which affects the strength and stability of mixed recycled construction-solid-waste aggregate [15,16]. Previous research studies have shown that brick aggregate content has a significant impact on the volume index of mixed recycled aggregate [17]; specifically, with the increase in brick aggregate content, the apparent density increases, and the maximum dry density decreases [18,19]. However, the effect of brick aggregate content on the mechanical performance of mixed recycled aggregates is still unclear. Furthermore, researchers have investigated the performance of mixed recycled aggregate [20,21], finding that the resilient modulus and deflection of construction-solid-waste material containing a small amount of recycled brick aggregate can meet the technical standard requirements [22,23].

At present, research on recycled aggregate mainly focuses on the evaluation of mechanical properties based on laboratory tests [24,25]. Although laboratory experiments can be easily performed, they cannot simulate field conditions well, which causes a discrepancy between laboratory design and subgrade construction [26]. The field compaction method for construction solid waste, the main cause of uneven settlement of the subgrade, is determined by the on-site engineers according to their experience [27]. Accurate control and scientific evaluation of compaction quality are effective ways to solve the above problems. The portable falling weight deflectometer (PFWD) is widely used in road engineering due to its advantages of being fast, accurate, lightweight, and environmentally friendly; the subgrade deflection and resilient modulus under different material compositions can be rapidly evaluated based on the pressure and displacement sensors of a PFWD [28].

Therefore, in order to evaluate the effect of brick aggregate content on the performance of recycled construction-solid-waste aggregate, a research method that innovatively combines laboratory experiments and field investigation was used in this study. The effect of brick aggregate content on the mechanical properties of recycled aggregate was analyzed through laboratory tests, and the optimal brick aggregate content and construction method were determined based on field measurement parameters. Our research results can provide theoretical support for the application of recycled brick–concrete-solid-waste aggregates in subgrade filling.

### 1.2. Research Plan

**Figure 1:**
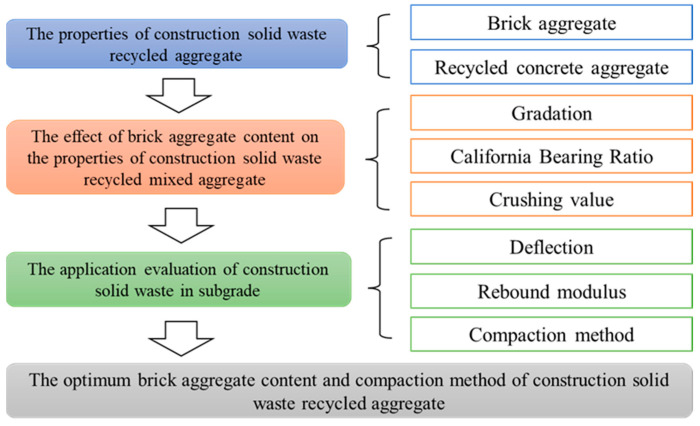
The research plan.

The purpose of this study was to evaluate the effect of brick aggregate content on the performance of recycled mixed construction-solid-waste aggregate and to select the optimal content and field compaction method. The research plan was executed as follows: Firstly, the properties of recycled mixed aggregate were tested to investigate the differences between brick aggregate and recycled concrete aggregate. Secondly, the effects of brick aggregate content on the gradation, CBR, and crushing value of mixed recycled aggregate were studied based on laboratory experiments. Finally, recycled construction-solid-waste aggregates with different brick aggregate contents were used for subgrade filling in field tests. The deflection and resilient modulus were tested by using a PFWD to evaluate the applicability of brick construction-solid-waste aggregate in subgrade filling, and the optimal brick aggregate content and compaction method were determined. The research plan is shown in Figure 1.

## 2. Materials and Methods

### 2.1. Properties of Construction-Solid-Waste Materials

**Figure 2:**
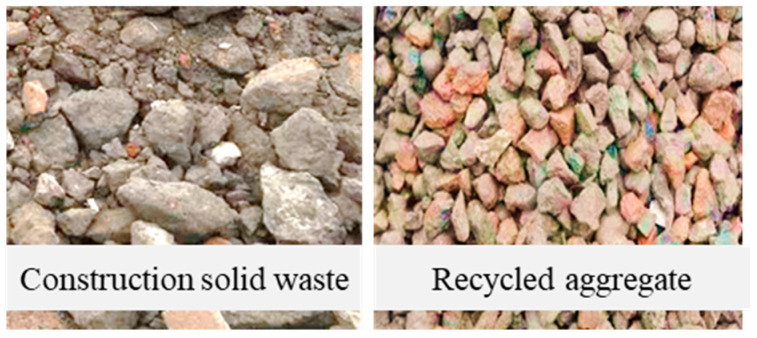
Recycled construction-solid-waste aggregate.

The jaw breaker produced by Yinhe Analytical Instrument Chemical Co., Ltd. (Hebi, China) was used to crush brick slag and waste concrete blocks and form recycled aggregates with a particle size range of 1.18–37.5 mm. To remove soil from the construction solid waste, the recycled aggregate was washed and dried for 3–4 h at a temperature of 100 °C, as shown in Figure 2.

Performance tests were conducted on the brick aggregate and the recycled concrete aggregate for recycled construction-solid-waste aggregate according to technical standard JTG E42-2005 [29], and a natural aggregate was used for comparison. As shown in Table 1, the water absorption rate of the recycled concrete aggregate was 5.30%, and the crushing value was 18.6%. The brick aggregate had low apparent density, and its water absorption rate and crushing value were higher than those of the recycled concrete aggregate. The results also indicate that the strength of the recycled construction-solid-waste aggregate was poor.

The brick slag was subjected to mechanical cutting and vibration during the crushing process. Due to the lower hardness of the brick slag, its edges and texture were more easily polished to produce brick slag powder with a particle size smaller than 1.18 mm. To analyze the effect of moisture content on the mechanical state of the brick slag powder, the plasticity characteristics were evaluated by using the liquid and plastic limit indices [30]. As shown in Table 2, the liquid limit (Wl) of the brick slag powder was 36.5%, which indicates a limit moisture content of 36.5% for the transition from the flowing state to the plastic state. The plastic limit (Wp) of the brick slag powder was 19.9%, which indicates a limit moisture content of 19.9% for the transition from the plastic state to the semi-solid state. The results also show that the plasticity index (Ip) of the brick slag powder was 17.5%, which indicates good plasticity.

### 2.2. Research Method

#### 2.2.1. Laboratory Tests

The recycled concrete aggregate and brick aggregate in the dry state were selected, and mixed aggregate samples were prepared according to the following six different contents of brick aggregate: 0%, 20%, 40%, 60%, 80%, and 100%. Two laboratory experiments were carried out to determine the samples’ bearing capacity and mechanical strength.

The California bearing ratio (CBR) index was used to evaluate the bearing capacity of the recycled mixed aggregates. The samples were prepared by using heavy compaction, according to technical standard JTG E40-2007 [31]. We considered three layers of compaction, and each layer was compacted 98 times, where 45 cm was selected as the falling height of the heavy compaction hammer. A 2.5 mm/min loading rate was applied until the sample settled at 25.4 mm [31]. The force and settlement data during the test were recorded to calculate the CBR.

In order to evaluate the firmness and wear resistance of the mixed recycled construction-solid-waste aggregate, the mechanical properties at the six brick aggregate content values were studied based on the crushing value index. The 9.5–13.2 mm aggregate samples were screened according to technical standard JTG E42-2005 [29]. Different proportions of brick aggregate and concrete recycled aggregate were prepared, and the crushing value test was carried out. In the test, we adopted the uniform-loading mode; once the total load reached 400 kN at 10 min, the pressure was stabilized for 5 s; then, the load was removed. Finally, an aggregate sample with a particle size smaller than 2.36 mm was employed to calculate the crushing value of the recycled mixed aggregate.

#### 2.2.2. Field Investigation

A test section of construction-solid-waste subgrade was constructed in Guangdong Province, China. Six different proportions of brick aggregate and recycled concrete aggregate were used to fill in the first part of the test section, and the deflection and resilient modulus of the field were measured by using a PFWD to evaluate the construction quality of mixed recycled aggregate with different proportions of construction solid waste. The optimal proportion of brick aggregate was used for field compaction in the second part of the test section, and the settlement characteristics of the subgrade were evaluated based on the thickness evolution characteristics of the fill layer.

## 3. Effect of Brick Aggregate Content on Performance of Mixed Recycled Aggregate

### 3.1. Effect of Brick Aggregate Content on CBR

**Figure 3:**
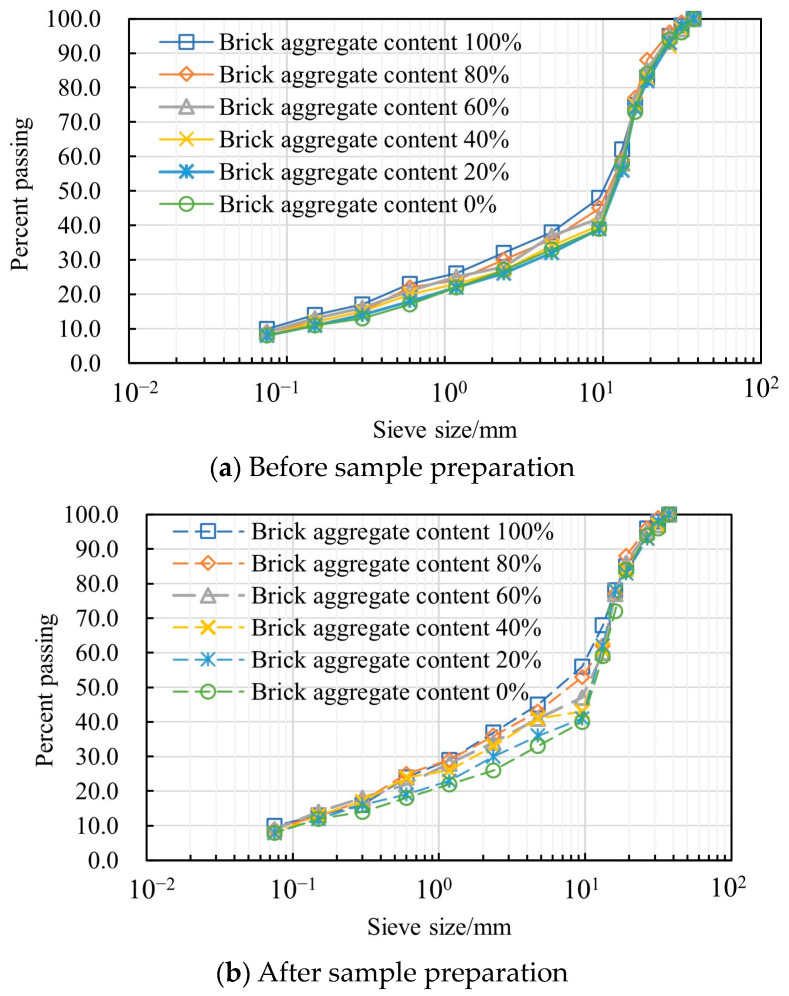
Gradation curves of recycled construction-solid-waste aggregate.

To analyze the stability of mixed recycled aggregate from construction solid waste during the preparation process of CBR specimens, screening tests were conducted on recycled aggregate with different brick aggregate contents, and the impact of brick aggregate crushing characteristics on the gradation of the mixed recycled aggregates during compaction was evaluated. Figure 3 shows the grading curves before and after sample preparation. It can be seen that, when the brick aggregate content was less than 80%, it had little influence on the gradation of the mixed recycled solid-waste aggregate before sample preparation, but, when it was greater than 80%, the pass rate of the 9.5 mm sieve was relatively high, which indicated that its gradation was relatively fine. It was not difficult to find that the gradation change was not evident in the compaction process when the brick aggregate content was 0. With an increase in brick aggregate content, the passing percent of the 9.5 mm sieve and the degree of change in gradation increased. When the brick aggregate content was 100%, the passing percentage of the 9.5 mm sieve increased by around 10%.

**Figure 4:**
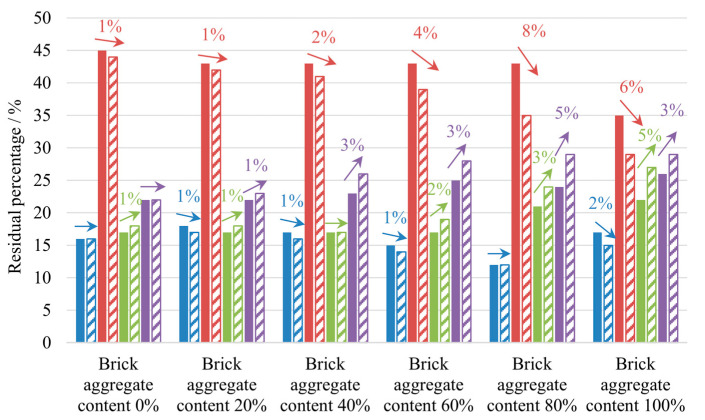
Aggregate content changes of samples with different particle sizes before and after compaction. Note: The blue, red, green, and purple columns represent the 19–37.5 mm, 9.5–19 mm, 1.18–9.5 mm, and 0.075–1.18 mm aggregates, respectively. Additionally, solid-filled columns indicate the residual percentages of the aggregate before compaction, and the diagonal-filled columns represent after compaction.

Each mixed recycled aggregate sample was divided into four parts according to particle size, i.e., between 19 and 37.5 mm, 9.5–19 mm, 1.18–9.5 mm, and 0.075–1.18 mm, and the residual percentage variation was analyzed. As shown in Figure 4, when the brick aggregate content was less than 40%, the residual percentage variation of aggregates with size greater than 19 mm was relatively small and that of aggregates with size less than 9.5 mm was relatively stable. On the contrary, with the increase in brick aggregate content, the residual percentage variation of aggregates with size less than 9.5 mm increased gradually and that of 9.5–19 mm recycled aggregate was reduced, which indicated that the aggregate was crushed to a higher degree during the compaction process. The results show that a greater content of brick aggregate led to a greater change in its residual percentage.

As a performance index, the CBR directly reflects the strength of mixed construction-solid-waste aggregate and has clear physical and mechanical significance. The load at a penetration depth of 5 mm was used to calculate the CBR. Four specimens for each brick aggregate content were prepared, and Table 3 shows the CBR results. It can be seen that the CBR continuously decreased with the increase in brick aggregate content, which indicates that the bearing capacity of the recycled aggregate was reduced by the brick aggregate. The CBR of the mixed recycled aggregate samples was also far higher than the requirement of 8% to meet the technology standard [32] when the content of brick aggregate was 100%. When the brick aggregate content was 0–40%, the reduction rate of the CBR was lower than that found for 40–100% brick aggregate content. The main reason for this finding is that, when the content of brick aggregate was less than 40%, there was enough high-strength concrete aggregate in the mixed recycled aggregate to form a stable skeleton structure. Therefore, with the increase in brick aggregate content, the strength of the mixed aggregate decreased slowly. Due to the stronger water absorption and expansion ability of brick aggregates, the water absorption rate and expansion amount of mixed recycled construction-solid-waste aggregates gradually increased with the increase in brick aggregate content. To ensure the bearing capacity of mixed recycled construction-solid-waste aggregate, the optimum content of brick aggregate is recommended to be 40%.

### 3.2. Effect of Brick Aggregate Content on Crushing Value

**Figure 5:**
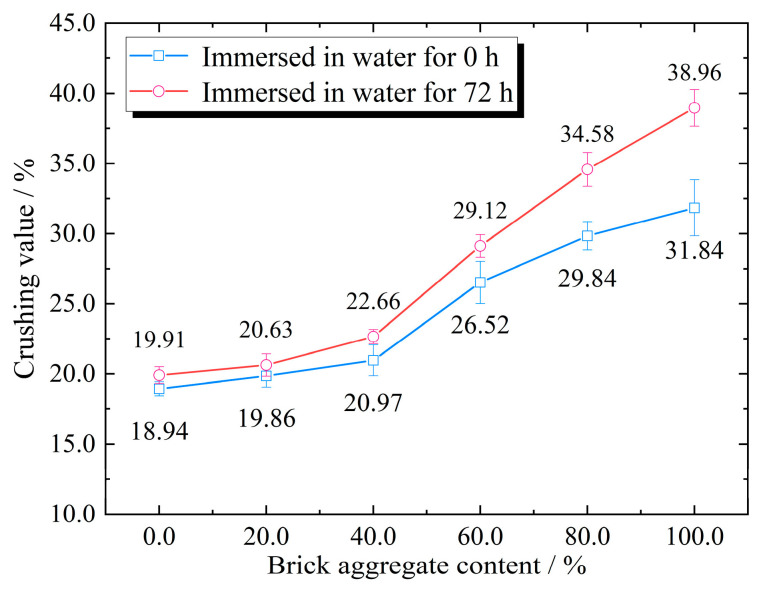
The crushing values of mixed recycled aggregates with different brick aggregate contents.

The crushing value was used to evaluate the resistance of aggregates to crushing under gradually increasing loads. A larger crushing value corresponded to more crushing of the aggregates under loading and a lower strength. The crushing performance of the mixed recycled aggregates with different brick aggregate contents was evaluated. As shown in Figure 5, the crushing value of the mixed recycled aggregate increased with the increase in brick aggregate content, and the greater the amount of brick aggregate, the greater the dispersion of the test results. When the brick aggregate content was 100%, the crushing value was 31.84%, which was 1.68 times the value found when the brick aggregate content was 0. The recycled aggregate’s crushing value sharply increased with an increase in brick aggregate content above 40%, and the crushing resistance decreased noticeably, which indicates that brick aggregate content above 40% has a greater impact on the overall strength of mixed recycled aggregate.

In previous studies, the water absorption characteristics of recycled aggregate have been evaluated [33,34], but the influence of water immersion on the mechanical properties of recycled aggregate has not. In this study, the crushing value test was conducted on the recycled aggregates after immersion. Because the properties of brick aggregate are easily affected by water, the soaking crushing value of the brick aggregate was analyzed after 72 h. As shown in Figure 5, when the mixed recycled aggregate did not contain brick aggregate, the soaking crushing value only increased by around 1%. Since the strength of brick aggregate is low after water absorption, the strength decrease in the samples was more evident when the content of brick aggregate was increased. Therefore, when the brick aggregate content was 100%, the soaking crushing value increased by around 7%, which indicates that a greater brick aggregate content corresponded with a greater influence of soaking on the crushing value. Measures should be taken to improve the moisture resistance of subgrade recycled aggregate. Consistent with the change characteristics of the crushing value in the dry state, when the content of brick aggregate was 40%, was that the crushing value had a noticeable inflection point. To ensure mixed recycled aggregate compressive strength, it is recommended that the content of brick aggregate not exceed 40%.

## 4. Field Compaction of Recycled Aggregate

### 4.1. Evaluation of Field Compaction Quality

In previous studies, laboratory tests and numerical simulations of recycled aggregates have been evaluated [35,36], but the influence of field parameters on pavement design has not been considered. In order to obtain the field parameters of recycled aggregates and analyze the applicability of recycled aggregates containing brick aggregate in subgrade filling, a test section of construction-solid-waste subgrade was constructed in Guangdong, China. The total length of the test section was 600 m, with a width of 28.5 m. The first 120 m of the test section was used to investigate the field deflection and resilient modulus of different brick aggregate contents, and the last 480 m was used to evaluate the compaction characteristics of the subgrade under different compaction methods. In order to reduce the interference of other factors during the experiments, the first part of the test section was divided into six different areas, each of which was 20 m in length and had a field compaction degree of 95%. The brick aggregate contents of the six areas were 0%, 20%, 40%, 60%, 80%, and 100%.

Subgrade construction quality was evaluated according to the index of deflection and the resilient modulus. At present, there are many methods for assessing deflection, among which the PFWD can simulate dynamic loads well and scientifically evaluate the dynamic deflection and resilient modulus generated under dynamic loads. In addition, the data measured by the sensor can be processed directly by a computer, which is faster and more accurate than human processing.

**Figure 6:**
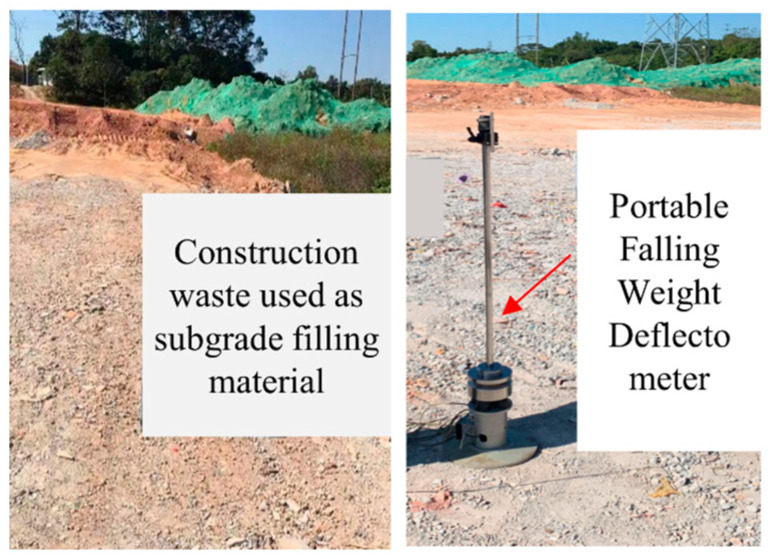
Recycled construction-solid-waste aggregate used for subgrade filling.

A PFWD was adopted for the field test, and a working plane with uniform and flat characteristics was selected, as shown in Figure 6. The test location was determined according to the method of uniform distribution. To reduce the boundary effect, starting from 2.85 m away from the edge of the subgrade, the test was conducted every 5.7 m in the horizontal direction and every 2 m in the vertical direction. A total of 300 points were considered in the test section.

**Figure 7:**
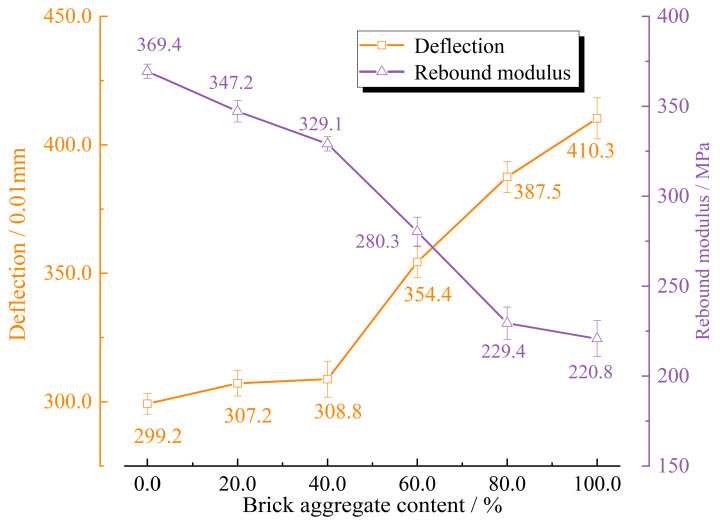
The results of the field test.

The resilient modulus and deflection of the test section with different brick slag contents were obtained, and the results are shown in Figure 7. It was found that, with the increase in brick slag content, the resilient modulus decreased, and the deflection increased. The reason for this finding is that the brick aggregate was crushed under the action of the dynamic load, and the greater its content, the higher the degree of crushing. In the crushing process, the relative displacement between the particles was large, which led to the increase in deflection and the decrease in resilient modulus.

There was discreteness in the field detection data of the test section, which gradually increased with the increase in brick slag content. In addition, it could also be seen that the deflection curve had a noticeable inflection point corresponding to the slag content of 40%, while the resilient modulus curve was smoother. When the content of brick slag was 40%, the measured deflection was 3.088 mm, and the resilient modulus was 329.1 MPa.

**Figure 8:**
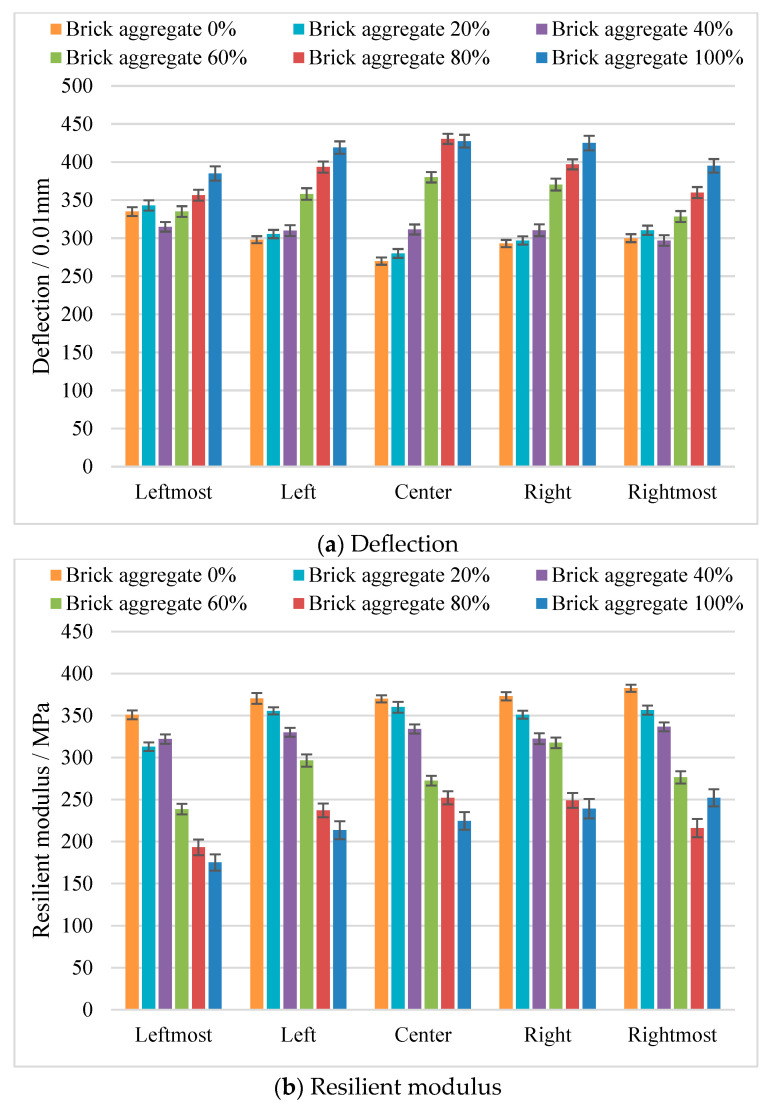
The results for different transverse locations.

In order to analyze the construction uniformity of the mixed recycled aggregates, we obtained the deflection and resilient modulus at different transverse locations at distances of 2.85 m, 8.55 m, 14.25 m, 19.95 m, and 25.65 m from the subgrade edge, representing the leftmost, slightly left, center, slightly right, and rightmost portions of the subgrade, respectively. As shown in Figure 8a, compared with the subgrade center, the deflection on both sides of the subgrade was slightly greater when the brick aggregate content was less than 20% and smaller when the brick aggregate content was greater than 60%; finally, when the brick aggregate content was 40%, the deflection at different distances from the subgrade edge was nearly the same, which indicates that the construction uniformity was high. The measured deflection value at the edge of the subgrade did not increase strictly with an increase in brick aggregate content, which indicates that the construction uniformity at the subgrade edge was more discrete.

The results of the resilient modulus at different transverse locations are shown in Figure 8b. It can be seen that, when the brick aggregate content was less than 20%, the resilient modulus was mostly consistent, except for the leftmost side of the subgrade. When the brick aggregate content was 40%, the uniformity of the subgrade was better. When the content of brick aggregate was greater than 60%, the resilient modulus at different positions was different. Finally, the resilient modulus in the center of the subgrade decreased with the increase in brick aggregate content, which indicates that the construction uniformity in the center of the subgrade was higher.

### 4.2. Compaction Parameters of Construction-Solid-Waste Subgrade

The compaction degree is a phenomenological volume parameter that does not directly reflect the stress and deformation characteristics of mixed recycled aggregate during the compaction process. To evaluate the effect of the compaction method on the thickness variation of the mixed recycled aggregates during the subgrade construction process, the second part of the test section was constructed with the mixed recycled aggregate with 40% brick aggregate content determined from the first experiment. The length was 480 m, and the filling thickness of the subgrade was 40 cm. A grid of 5.7 m × 5.87 m was delineated within the fill area, and mixed recycled aggregate (approximately 12 m^3^) was poured into each grid to prevent its segregation. Finally, the mixed recycled aggregate materials in different grids were pressed together to avoid large voids as much as possible.

A compacting machine, a Xugong 22 t vibratory roller produced by XCMG Construction Machinery Co., Ltd. (Xuzhou, China), was used for compaction from the edge of the subgrade to the center, and the speed of the roller was set to 3 km/h. The initial compaction stage included three passes of the compaction roller, followed by static pressure, weak vibration, and strong vibration. The main compaction stage was from the fourth to the eighth pass, and the ninth pass represented the final compaction stage, after which static pressure was applied. Four different methods were used for field compaction during the main compaction stage, as shown in Table 4, where each was employed in a test section length of 120 m. The LyCRA TCR402 total station produced by Eurasia Measuring System Equipment Co., Ltd. (Shanghai, China) was used to detect the height of the subgrade after each roller pass during the main compaction stage. The thickness of the fill layer was calculated based on the height difference, and the changes in fill thickness with different compaction methods were analyzed.

**Figure 9:**
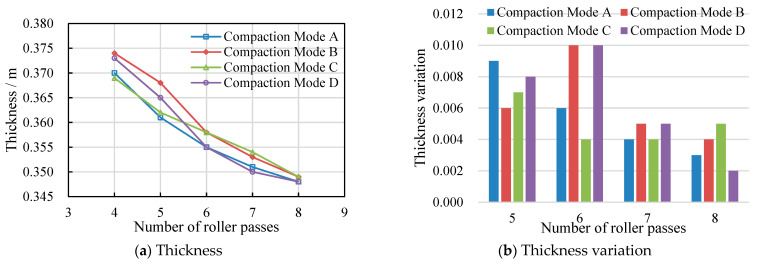
Thickness results under different compaction methods.

The results of fill layer thickness under different compaction methods are shown in Figure 9a. It can be seen that the fill thickness continued to decrease with the increase in roller passes in the main compaction stage. The significant difference in the thickness variation under different compaction methods indicates that the compaction method had an impact on fill thickness during the compaction process. Although the final thickness of the fill layer was around 0.35 m after the main compaction stage, the variation in thickness under different compaction methods at the eighth pass was inconsistent, which indicates that the stability of the final subgrade was different.

The variation in fill thickness after each pass was calculated. As shown in Figure 9b, when Mode A was applied in the process of compaction, the variation in layer thickness decreased with the increase in the number of passes, which indicates that it became more and more stable. When Modes B and D were applied, the layer thickness first increased and then decreased with the increase in passes, and the layer thickness changed the most after the sixth pass. The layer thickness variation under Modes D and B was inconsistent after the fifth and eighth passes, where Mode D caused a greater change in layer thickness after the fifth pass but a smaller one after the eighth pass of only 2 mm. The layer thickness under Mode C showed the smallest change after the sixth pass and then did not decrease with the increase in passes. The results of the thickness change show that the construction effects of Modes A and D were better.

**Figure 10:**
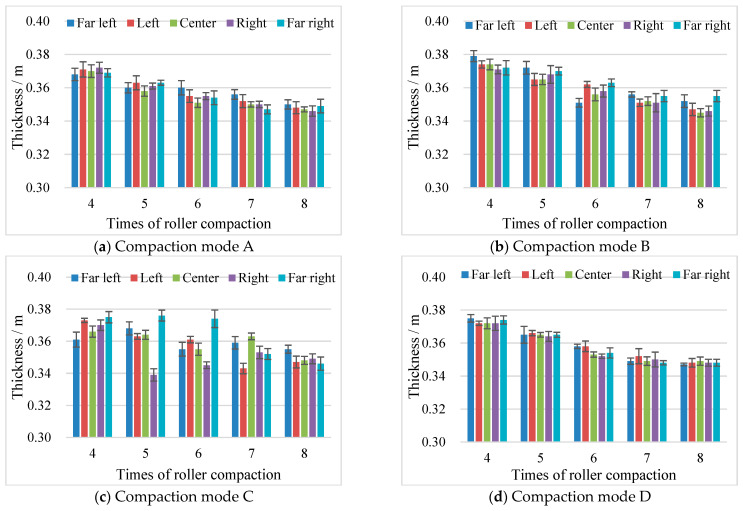
Thickness results for different transverse locations (The error bars represent standard deviation).

The layer thickness variation at different transverse locations during the main compaction stage was analyzed. As shown in Figure 10, due to the difference in the properties of the recycled aggregates, there were a few void structures in the materials, so the measured data at each location were not completely consistent. The test results obtained under Modes B and C differ greatly at different transverse positions, which indicates that uniformity was poor for both compaction methods. There was a significant difference in thickness between the edge and the middle of the fill layer under Mode A after the seventh and eighth passes. The results for five different transverse locations obtained under Mode D are mostly consistent. Moreover, the layer thickness varied less after the eighth pass, which indicates that the compaction quality obtained with Mode D was high. Therefore, it is recommended to use a 22 t roller for five passes (two weak vibrations + two strong vibrations + one weak vibration) at a speed of 3 km/h as the compaction method for recycled construction-solid-waste aggregate subgrade filling.

## 5. Conclusions

(1) A brick aggregate content less than 80% had little effect on the gradation of mixed recycled aggregate. During the compaction process, the recycled aggregate with a particle size of 9.5 mm to 19 mm was crushed to a higher degree, and a larger amount of brick aggregate led to a higher degree of crushing.
(2) With an increase in brick aggregate content, the CBR decreased continuously, and when the content was 0–40%; the reduction rate of the CBR was smaller than that found for 40–100% content. However, even when the content of brick aggregate was 100%, the CBR was much higher than the technology standard requirement of 8%.
(3) The crushing value of the mixed recycled aggregates increased with an increase in brick aggregate content. The larger the value of the latter, the greater the dispersion of the test results and the greater the influence of the soaking crushing value. When the brick aggregate content was greater than 40%, the crushing value of the recycled aggregates increased sharply, and the crushing resistance decreased noticeably.
(4) With an increase in brick slag content, the resilience modulus decreased, the deflection increased, and the brick aggregate content affected the uniformity of compaction. When the brick aggregate content was 40%, the construction uniformity was good.
(5) The recommended ratio of brick aggregate to recycled concrete aggregate for mixed recycled construction-solid-waste aggregate is 40% to 60%. The results show that the subgrade filling construction method, whereby a 22 t roller is used for compaction for five passes (two weak vibrations + two strong vibrations + one weak vibration) at a speed of 3 km/h in the main compaction stage, achieves better construction quality.
(6) Due to limitations in engineering practices, only recycled aggregates with six different brick aggregate contents were selected for performance evaluation in this study. Future research could continue to analyze the influence of subgrade structural parameters on the performance of recycled aggregates from construction solid waste based on further field testing.

## Figures and Tables

**Table 1:** The properties of the recycled construction-solid-waste aggregate.

Material	Apparent Density (kg·m^−3^)	Water Absorption Rate (%)	Crushing Value (%)	Moisture Content (%)
Brick aggregate	2038	12.70	42.3	4.03
Concrete aggregate	2580	5.30	18.6	2.50
Natural aggregate	2863	1.01	13.2	1.23

**Table 2:** Results of liquid and plastic limit tests.

Material	Liquid Limit (W_l_) (%)	Plastic Limit (W_p_) (%)	Plasticity Index (I_p_) (%)
Brick slag powder	36.5	19.9	16.6

**Table 3:** CBR test results of recycled aggregate for construction solid waste.

Content of Brick Aggregate (%)	CBR (%)	Requirement of CBR (%)	Expansion Ratio (%)	Water Absorption Rate (%)
0	36.1	8	0.017	7.6
20	34.2		0.017	10.0
40	32.8		0.021	11.8
60	28.4		0.031	10.6
80	24.7		0.038	12.8
100	18.4		0.044	13.1

**Table 4:** Construction methods in the repressing stage.

Mode	A	B	C	D
Compaction method	2 strong vibrations + 3 weak vibrations	3 weak vibrations + 2 strong vibrations	1 strong vibration + 3 weak vibrations + 1 strong vibration	2 weak vibrations + 2 strong vibrations + 1 weak vibration

## Data Availability

The original contributions presented in the study are included in the article, further inquiries can be directed to the corresponding author.
